# An *in silico* Framework of Cartilage Degeneration That Integrates Fibril Reorientation and Degradation Along With Altered Hydration and Fixed Charge Density Loss

**DOI:** 10.3389/fbioe.2021.680257

**Published:** 2021-06-22

**Authors:** Seyed Ali Elahi, Petri Tanska, Rami K. Korhonen, Rik Lories, Nele Famaey, Ilse Jonkers

**Affiliations:** ^1^Department of Movement Sciences, KU Leuven, Leuven, Belgium; ^2^Mechanical Engineering Department, KU Leuven, Leuven, Belgium; ^3^Department of Applied Physics, University of Eastern Finland, Kuopio, Finland; ^4^Department of Development and Regeneration, Skeletal Biology and Engineering Research Center, Division of Rheumatology, KU Leuven and University Hospitals Leuven, Leuven, Belgium

**Keywords:** mechanobiological modeling, finite element method, cartilage degeneration, osteoarthritis, adaptive modeling, *in silico* techniques, articular cartilage, regulatory algorithm

## Abstract

Injurious mechanical loading of articular cartilage and associated lesions compromise the mechanical and structural integrity of joints and contribute to the onset and progression of cartilage degeneration leading to osteoarthritis (OA). Despite extensive *in vitro* and *in vivo* research, it remains unclear how the changes in cartilage composition and structure that occur during cartilage degeneration after injury, interact. Recently, *in silico* techniques provide a unique integrated platform to investigate the causal mechanisms by which the local mechanical environment of injured cartilage drives cartilage degeneration. Here, we introduce a novel integrated Cartilage Adaptive REorientation Degeneration (CARED) algorithm to predict the interaction between degenerative variations in main cartilage constituents, namely collagen fibril disorganization and degradation, proteoglycan (PG) loss, and change in water content. The algorithm iteratively interacts with a finite element (FE) model of a cartilage explant, with and without variable depth to full-thickness defects. In these FE models, intact and injured explants were subjected to normal (2 MPa unconfined compression in 0.1 s) and injurious mechanical loading (4 MPa unconfined compression in 0.1 s). Depending on the mechanical response of the FE model, the collagen fibril orientation and density, PG and water content were iteratively updated. In the CARED model, fixed charge density (FCD) loss and increased water content were related to decrease in PG content. Our model predictions were consistent with earlier experimental studies. In the intact explant model, minimal degenerative changes were observed under normal loading, while the injurious loading caused a reorientation of collagen fibrils toward the direction perpendicular to the surface, intense collagen degradation at the surface, and intense PG loss in the superficial and middle zones. In the injured explant models, normal loading induced intense collagen degradation, collagen reorientation, and PG depletion both on the surface and around the lesion. Our results confirm that the cartilage lesion depth is a crucial parameter affecting tissue degeneration, even under physiological loading conditions. The results suggest that potential fibril reorientation might prevent or slow down fibril degradation under conditions in which the tissue mechanical homeostasis is perturbed like the presence of defects or injurious loading.

## Introduction

Osteoarthritis (OA) is a complex multi-faceted joint disease of which articular cartilage degeneration is a hallmark. OA is a prevalent disease in the elderly, but younger patients can be affected by mechanically induced OA due to an injury or chronic overloading of the tissue (e.g., due to sports activities) (Mukherjee et al., 2020). OA compromises the biological and mechanical integrity of articular cartilage, whose main role is to reduce the friction between articulating bone surfaces and transmit loads to the underlying subchondral bone (Da Silva et al., 2009; Eskelinen et al., 2019). Despite extensive studies, as detailed in the following paragraphs, so far, the mechanisms behind mechanically induced OA are not fully understood. The focus of this paper is to propose an integrated *in silico* cartilage degeneration model including key features of cartilage damage. The model predictions are compared with previous experimental observations on the role of injurious mechanical loading and the presence of focal defects in cartilage degeneration.

### Articular Cartilage Composition

Articular cartilage is an avascular tissue composed of chondrocytes embedded within their self-produced extracellular matrix (ECM). The biphasic ECM is composed of water and a solid phase (Mohammadi et al., 2013). The main constituents of the solid phase are collagen fibrils and proteoglycans (PGs). The collagen fibrils form an arcade-shaped fibril network which is organized into three layers known as superficial, middle and deep zones. The PGs control water content through variations in the hydrophilic negatively charged glycosaminoglycan (GAG) content, these produce a negative fixed charge density (FCD) within the tissue (Roughley and Lee, 1994; Hosseini et al., 2014; Orozco et al., 2018). The FCD causes osmotic pressure differences within the tissue and subsequently, cartilage swelling. The collagen network resists the swelling through the tensile strength of the collagen fibrils and prevents the extrusion of PGs from the ECM during interstitial fluid flow (Julkunen et al., 2013; Gardiner et al., 2016). In the macro-scale, this swelling behavior is critical in resisting compressive loads and therefore maintaining the unique mechanical properties of cartilage (Fox et al., 2009).

### Mechanically Induced OA Onset and Progression

Cartilage homeostasis maintains the structural properties and unique mechanical behavior of the tissue through sustained ECM synthesis. Injurious loading to the articular cartilage and consequent lesions (Dulay et al., 2015) may change the stress and strain distribution within the tissue (Wilson et al., 2006a; Speirs et al., 2014; Ferizi et al., 2017; Tanska et al., 2018). These alternations are often associated with or followed by chondrocyte dedifferentiation and apoptosis, PG depletion, as well as collagen fibril disorganization and degradation (Loening et al., 1999; Horton et al., 2006; Wilson et al., 2006a; Ferizi et al., 2017; Tanska et al., 2018). Local PG depletion will cause FCD loss and consequently FCD will attract less water into the tissue. Conversely, loss (and disorganization) of solid contents due to PG depletion increases tissue hydration (Sah et al., 1991; Setton et al., 1999; Men et al., 2017). An increase in tissue hydration was found to be a major contributor to collagen network disorganization (Saarakkala et al., 2010) and a decrease in tissue stiffness (Buckwalter, 1992). Therefore, a chain of degenerative mechanisms (i.e., fibril network disorganization, PG depletion and fibril degradation) is suggested to underly OA development following injurious cartilage loading, however, their exact interactions and in particular the roles of fibril disorganization and increased tissue hydration are not clear.

Understanding the various mechanisms behind the onset and progression of mechanically induced OA and their interactions is crucial to elucidate their role and optimize treatment methods. However, *in vivo* and *in vitro* studies face several limitations to evaluate the interactive roles of collagen disorganization and degradation, FCD loss and increase in tissue hydration in OA onset and progression. These are related to limited access to samples and test data, the need for specific experimental facilities, and high costs. Indeed, in *in vivo* and *in vitro* experiments, multiple processes occur simultaneously and their mutual influence and unique contribution to OA onset and progression cannot be isolated.

### *In silico* Models to Predict OA Onset and Progression

*In silico* models provide a unique platform to incorporate insights from *in vivo* and *in vitro* experiments. These models leverage enhanced understanding of the local mechanical environment in cartilage tissue under injurious loading and around structural defects as well as its contribution to cartilage damage. To this end, several *in silico* models were introduced in the literature (Keenan et al., 2013; Wu et al., 2016; Koh et al., 2019; Wang et al., 2019). Among the proposed models, a fibril-reinforced poro-viscoelastic swelling (FRPVES) finite element (FE) model introduced by Wilson et al. (2005) accounts for different ECM constituents (i.e., collagen content, fibril orientation, PG content, and water content) and therefore allows studying the effect of variations in cartilage composition due to the altered mechanical environment. To simulate cartilage degeneration and load-dependent changes in the contents of ECM constituents, several adaptive algorithms have previously been introduced in the FRPVES model. These adaptive algorithms have been used to predict the individual effect of collagen network disorganization (Wilson et al., 2006a; Tanska et al., 2018), PG depletion (Orozco et al., 2018, 2020; Eskelinen et al., 2019), collagen degradation (Wilson et al., 2006b; Mononen et al., 2016; Liukkonen et al., 2017), and combined PG depletion and collagen degradation (Julkunen et al., 2013; Quiroga et al., 2017; Mononen et al., 2018). Aside from a previously proposed model that predicts the individual effect of PG depletion in cartilage degeneration through a decrease in FCD content (Orozco et al., 2018, 2020; Eskelinen et al., 2019), all other adaptive cartilage degeneration models predict tissue degradation through a decrease in material properties associated to PG or collagen content but do not change the introduced PG and collagen contents to the model.

Although these studies provide insights into local degenerative changes in cartilage tissue, they fail to predict changes in the contents of cartilage constituents and their isolated role in different degeneration mechanisms as well as their interactions. More specifically, existing cartilage degeneration algorithms lack biofidelity as they fail to (i) predict the changes in the contents of cartilage constituents (i.e., collagen and water contents) due to degeneration, (ii) account for experimentally observed effect of fibril disorganization (Makela et al., 2012), in combination with other degenerative mechanisms, and (iii) model the local increase in tissue hydration as a consequence of cartilage degeneration. The integration of these different degeneration mechanisms (in particular collagen fibril reorientation and degradation, FCD loss and increase in water content due to PG depletion) and their interaction into an integrated FE framework would allow a more mechanistic insights into cartilage degeneration. This would fulfill a currently unmet need clearly identified in literature (Wilson et al., 2006a; Tanska et al., 2018; Eskelinen et al., 2019; Mukherjee et al., 2020).

In this study, for the first time, we present an integrated adaptive FE framework that predicts the cartilage degenerative behavior through variations in cartilage constituents (i.e., collagen, FCD and water contents). In this framework, the previously developed algorithms for collagen fibril reorientation (Wilson et al., 2006a; Tanska et al., 2018) and degradation (Valentín et al., 2013; Famaey et al., 2018) were adapted and integrated with a novel PG depletion algorithm to predict the interactive effect of different degenerative mechanisms in cartilage degeneration: the collagen fibril degradation algorithm was adopted from an existing arterial degradation model (Valentín et al., 2013) and implemented to predict the decrease in collagen content due to cartilage degeneration. Furthermore, PG depletion was coupled to a decrease in the FCD content and a consequent increase in tissue hydration as part of the cartilage degeneration processes. This is in contrast to previous implementations where PG depletion was primarily modeled through a decrease in FCD content (Orozco et al., 2018, 2020; Eskelinen et al., 2019) or variations in cartilage material properties (Julkunen et al., 2013; Quiroga et al., 2017; Mononen et al., 2018). The performance of the novel Cartilage Adaptive REorienetation Degeneration (CARED) algorithm was evaluated with FE models of (i) an intact cartilage explant under normal loading, (ii) an intact cartilage explant under injurious loading, and (iii) cartilage explants with focal defects in accordance with the International Cartilage Regeneration and Joint Preservation Society (ICRS) grades 1, 2, and 3 under normal loading. This provides unique insights into the complex cascade/interactions of the different processes that affect the cartilage constituents and drive cartilage degeneration following injurious loading and cartilage injury.

## Materials and Methods

### Finite Element Modeling

**FIGURE 1:**
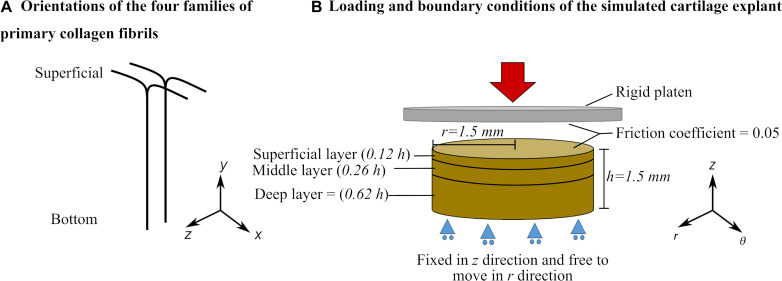
**(A)** Primary collagen fibril orientation and **(B)** Geometry, loading, boundary conditions and layers of the cartilage explant defined in the finite element model (see Supplementary Material for details about cartilage layers and depth-dependent properties).

A 3D description of the FRPVES material with Donnan osmotic swelling (Wilson et al., 2005; Eskelinen et al., 2019) was used to simulate the mechanics of articular cartilage. The structural, compositional and material parameters of the FRPVES model for healthy bovine articular cartilage were adopted from Tanska et al. (2018) and Eskelinen et al. (2019). Detailed descriptions of the material model, initial cartilage composition and parameters are provided in Supplementary Table 1. In the 3D description of the FRPVES model, the collagen network consists of four arcade-shaped primary fibrils and 13 randomly oriented secondary fibrils. Experimental observations show that the primary fibrils at the superficial layer are oriented in two directions in most parts of the articular cartilage tissue (Clark, 1985; Mononen et al., 2012). Therefore, the primary fibrils were oriented in two directions forming split-lines at the model surface (+x and –x directions in Figure 1A).

**FIGURE 2:**
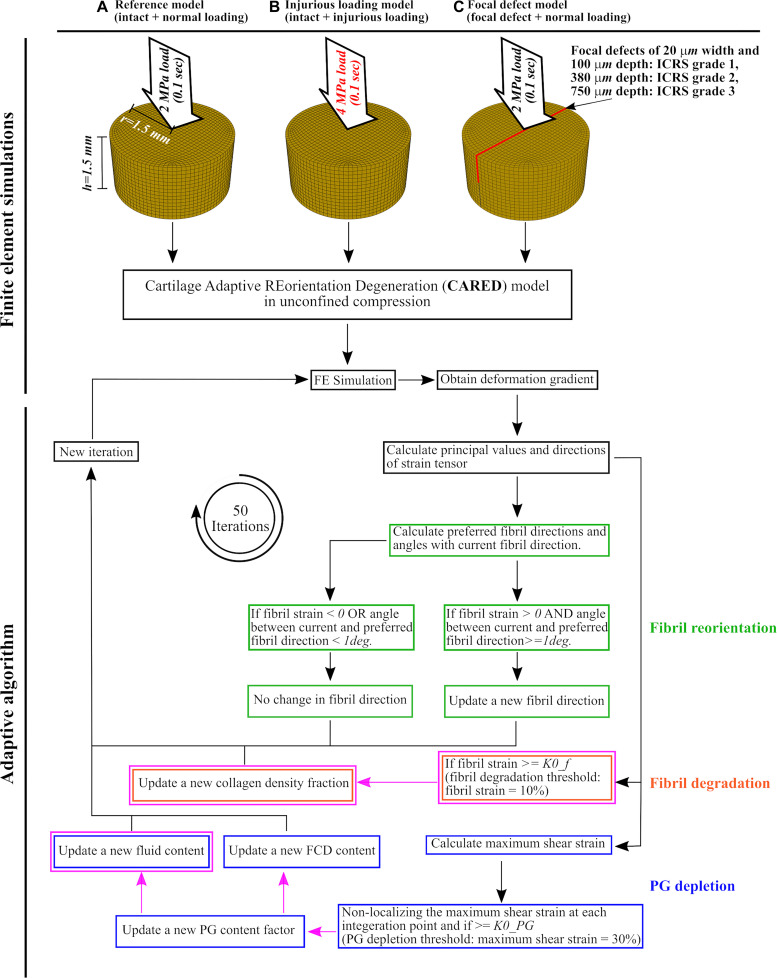
Cartilage explant geometries and applied loadings for the finite element model and the adaptive algorithm: **(A)** the reference model, **(B)** the injurious loading model, and **(C)** the focal defect models of ICRS grades 1, 2, and 3. After finite element simulation, the obtained deformation gradient was used to calculate principal values and directions of strain tensor at each element. These values were used to calculate the fibril reorientation [green frames, adopted from Tanska et al. (2018)], fibril degradation [orange frames, adopted from the studies on arterial tissue (Valentín et al., 2013; Famaey et al., 2018) and changed for cartilage] and PG depletion [blue frames, partially adopted from Eskelinen et al. (2019) and Quiroga et al. (2017)]. The magenta frames and arrows highlight the novelties in (i) adapting the collagen degeneration theory from arterial tissue to cartilage, (ii) using a non-localization theory for PG depletion, (iii) introducing a new algorithm for the increase in tissue hydration, and (iv) relating hydration variations and FCD loss to PG depletion. The definitions of preferred fibril directions and the angle between current and preferred fibril direction can be found in section “Collagen Fibril Reorientation Algorithm”.

Three groups of FE models of cartilage explants were created (Figure 2): (Figure 2A) reference model: intact explant with normal gait loading assumed to be a 2 MPa ramp load in 0.1 s (Kłodowski et al., 2016; Tanska et al., 2018; Eskelinen et al., 2019), (Figure 2B) injurious loading model: intact explant with injurious loading assumed to be a 4 MPa ramp load in 0.1 s (Loening et al., 1999, 2000; Quinn et al., 2001), and (Figure 2C) injury model: three explant models each included a 20 μm wide and either a 100, 380, or 750 μm deep lesion throughout the explant (Tanska et al., 2018) mimicking the ICRS defect grades 1, 2, and 3, respectively. Explants of this group were subjected to normal gait loading response (approximated with 2 MPa ramp load in 0.1 s).

The FE models of cartilage explants were created with cylindrical geometries (thickness *h* = 1.5 mm and radius *r* = 1.5 mm, Figure 1B). The bottom surface of the explant was restricted against vertical translation but allowed radial expansion. First, the cartilage explant was allowed to swell freely to reach mechanical equilibrium. During the free swelling step, the fluid was able to flow through the side and top surfaces (pore pressure = 0). The free swelling step was followed by a compression loading that was applied using a rigid platen on the top surface of the explant. During the compression step, the fluid flow through the top surface was restricted. The friction coefficient between the rigid platen and explant surface was set to 0.05 to simulate cartilage-to-cartilage contact (McCann et al., 2009; Eskelinen et al., 2019).

Intact cartilage explants and cartilage explants with focal defects corresponding to ICRS grades 1, 2, and 3 were meshed by 12,960, 15,176, 14,816, and 14,100 linear pore pressure continuum elements (element type C3D8P), respectively. Mesh convergence was ensured by modeling cartilage explant using half, twice, and four times of the selected element numbers. Simulations with higher mesh densities showed no substantial differences in deformation gradient distributions (the FE simulation output used in the adaptive algorithm of section “CARED Model”).

### CARED Model

To predict the interaction between collagen fibril disorganization and degradation, as well as PG depletion, a novel integrated FE-based framework (CARED model) was introduced. The CARED model input parameter is the deformation gradient tensor (***F***) of the FE simulation that iteratively determines the mechanically induced fibril reorientation and degradation, as well as the PG depletion in the articular cartilage. In this study, the reorientation and degenerations were assumed to take place with respect to the undeformed state, and thus, the strain in the cartilage tissue was evaluated in a Lagrangian frame from the Green–Lagrangian strain tensor ***E*** (Tanska et al., 2018):

(1) $$E=\frac{1}{2}\left(C-I\right),$$

where ***C*** is the right Cauchy-Green strain tensor:

(2) $$C=F^{T}\cdot F.$$

The principal values (λ_*j*_) and directions (*n_j_*) of ***E*** were calculated and used in the reorientation and degeneration algorithms.

Figure 2 shows a general overview of the CARED adaptive model, where the fibril reorientation, fibril degradation, and PG depletion algorithms are highlighted with green, orange, and blue frames respectively. Besides the novelty regarding integrating a fibril reorientation algorithm with collagen degradation and PG depletion mechanisms, the magenta frames and arrows in Figure 2 highlight the novelties in (i) adapting the collagen degeneration theory from arterial tissue (Valentín et al., 2013; Famaey et al., 2018) to cartilage, (ii) using a non-localization theory for PG depletion, (iii) introducing a novel algorithm for the increase in tissue hydration, and (iv) relating the increase in tissue hydration and FCD loss to PG depletion (details are given in the following paragraphs). The fibril reorientation, FCD loss, and non-localization theories were adopted from previous studies: (Wilson et al., 2006a; Tanska et al., 2018), (Orozco et al., 2018; Eskelinen et al., 2019) and (Quiroga et al., 2017), respectively. The procedure was repeated in 50 consecutive iterations of arbitrary time after which the reorientation and degeneration values reached an equilibrium.

#### Collagen Fibril Reorientation Algorithm

A previously developed collagen fibril reorientation algorithm was integrated to predict the fibril network disorganization in mechanically altered cartilage tissue, as observed experimentally (Makela et al., 2012). This algorithm assumes that collagen fibrils align according to a tensile strain direction, also confirmed by other computational and experimental studies (Driessen et al., 2005; Makela et al., 2012; Nagel and Kelly, 2013). This algorithm was initially introduced for arterial tissue by Driessen et al. (2003) and adapted for cartilage by Wilson et al. (2006a) and Tanska et al. (2018).

In our CARED model, we integrated the reorientation algorithm from Tanska et al. (2018). This algorithm proposes that the fibrils reorient toward a preferred fibril direction:

(3) $$e_{\text{p}}=\frac{{\text{g}}_{1}{\text{n}}_{1}\pm {\text{g}}_{2}{\text{n}}_{2}\pm {\text{g}}_{3}{\text{n}}_{3}}{\sqrt{{\text{g}}_{1}^{2}+{\text{g}}_{2}^{2}+{\text{g}}_{3}^{2}}},$$

where *n_j_* are the principal strain directions and *g_j_* are the functions of principal values of Green–Lagrangian strain tensor:

(4) $$\left\{ \begin{array}{c} g_{\text{j}}=\lambda_{\text{j}},\lambda_{\text{j}}>0\\ g_{\text{j}}=0,\lambda_{\text{j}}\leq 0 \end{array}\right.$$

Therefore, only positive principal strains contributed to the fibril reorientation. Equation (3) may result in up to four preferred fibril directions, among which the closest preferred direction to the current fibril direction (**e**_f_*i*_) was used to determine the fibril reorientation around a rotation axis defined as:

(5) $$e_{\text{r}}=\frac{{\text{e}}_{{\text{f}}_{\text{\_i}}}\times {\text{e}}_{\text{p}}}{∥{\text{e}}_{{\text{f}}_{\text{\_i}}}\times {\text{e}}_{\text{p}}∥}$$

The new fibril direction was calculated as:

(6) $${\text{e}}_{\text{f}\_i+1}=\text{exp}\left(\frac{d\theta }{dt}\hat{K}\right){\text{e}}_{\text{f}\_i},$$

where $\hat{K}$ is cross-product matrix of ***e***_*r*_ and $\frac{d\theta }{dt}$ is the angular velocity of reorientation defined as:

(7) $$\frac{d\theta }{dt}=\kappa \alpha =\kappa \text{arccos}∥{\text{e}}_{\text{f}\_i}\cdot {\text{e}}_{\text{p}}∥,$$

where α is the angle between the current (***e***_f_*i*_) and preferred (***e***_p_) fibril directions and κ is the reorientation rate, which was defined as:

(8) $$\left\{ \begin{array}{c} \kappa =0.3,\mathrm{if}\alpha \geq 1^{\circ }\text{and}\varepsilon_{\text{f}}>0\\ \kappa =0,\text{if}\alpha <1^{\circ }\text{or}\varepsilon_{\text{f}}\leq 0 \end{array}\right.,$$

i.e., the fibril reorientation was only allowed if α ≥ 1° and fibril experiences tension (ε_f_ is the strain in fibril direction- see Supplementary Material). The value of 0.3 was selected based on previous studies (Wilson et al., 2006a; Tanska et al., 2018). In this study, this value is a computational parameter without a physical time scale and it controls the reorientation rate for optimal convergence.

To optimize model convergence, the aforementioned calculations were performed for one of the four primary fibrils. One of the remaining primary fibrils was assumed to reorient in the same direction as the first fibril and the two others were assumed to reorient symmetrical to the calculated reorientation (symmetry was calculated concerning the *y-z* plane in Figure 1A).

#### Collagen Fibril Degradation Algorithm

To develop an algorithm for collagen degradation, a theory originally introduced for arterial tissue was used (Valentín et al., 2013; Famaey et al., 2018). The theory suggests that the contribution of collagen fibril in tissue stiffness in the next iteration (*Coll*_contrib,*i*1_) can be obtained from its contribution to the current iteration (*Coll*_contrib,*i*_) as follows:

(9) $$Coll_{\text{contrib},i+1}=Coll_{\text{contrib},i}D_{\text{coll}},$$

where *D*_*coll*_ is the collagen degradation rate calculated in relation to a damage function (β) and a material damage parameter (*m*_*coll*_):

(10) $$D_{\text{coll}}=1-\text{exp}\left(-\frac{\beta }{m_{\text{coll}}}\right)$$

Computational findings suggest that the tensile stimulus to the collagen network needs to be considered in the adaptive modeling of the collagen fibril degradation in cartilage (Wilson et al., 2006b; Hosseini et al., 2014; Tanska et al., 2018). Therefore, tensile strain in the fibril was used as a threshold for the fibril degradation (i.e., the fibril degradation occurs if ε_f_ > *K*_0,f_) (Hosseini et al., 2014; Famaey et al., 2018). The threshold was assumed to be *K*_0,f_ = 10% (Famaey et al., 2018). To adapt the collagen degradation model for cartilage, β was estimated with:

(11) $$\beta =\left|\varepsilon_{\text{f}}-K_{0,\text{f}}\right|,$$

and for simplicity and to reduce the number of model parameters it was assumed that *m*_coll_ = 1. In the FRPVES model, the contribution of collagen fibrils to tissue stiffness was assumed with depth-dependent collagen fibril density (*Coll*_contrib,*i*_ = ρ_z,*i*_- see Supplementary Table 1). Therefore, the collagen fibril degradation theory for cartilage tissue was calculated as:

(12) $$\rho_{\text{z},i+1}=\left\{ \begin{array}{c} \rho_{\text{z},i}\;\text{if}\varepsilon_{\text{f}}\leq K_{0,\text{f}}\\ \left[1-\exp\left(-\left|\varepsilon_{\text{f}}-K_{0,\text{f}}\right|\right)\right]\rho_{z,i}\text{if}\varepsilon_{\text{f}}>K_{0,\text{f}} \end{array}\right.$$

The greater the ε_f_, the collagen fibril density decreases more with consecutive loading iterations of arbitrary time. More details about the collagen fibril density parameter can be found in Supplementary Material.

#### Proteoglycan Depletion Algorithm

We implemented a PG depletion algorithm developed by Eskelinen et al. (2019). They conducted a parameter sensitivity analysis study on the different threshold parameters and values for adaptive FE modeling of the PG depletion in articular cartilage. The results show that maximum shear strain (Equation 13) with a threshold value of *K*_0,PG_ = 30% can predict the FCD loss in cartilage explants with focal defect most accurately compared to experiments.

(13) $$\varepsilon_{\text{max}}=\text{max}\left\{\left|\varepsilon_{\text{p},1}-\varepsilon_{\text{p},2}\right|,\left|\varepsilon_{\text{p},1}-\varepsilon_{\text{p},3}\right|,\left|\varepsilon_{\text{p},2}-\varepsilon_{\text{p},3}\right|\right\},$$

where ε_p,1_, ε_p,2_ and ε_p,3_ are the principal strains of Green–Lagrangian strain tensor ***E***.

Within the CARED model, a non-localized version of the PG depletion algorithm proposed by Eskelinen et al. (2019) was used, as mesh-dependent localization of damage is a known problem in mechanical modeling of tissue damage (Hosseini et al., 2014; Mukherjee et al., 2020). In damage theories, this is solved by using non-localizing methods for the damage evolution. First, ε_max_ was non-localized (ε_max, nl_) using a previously introduced non-localizing theory for cartilage degeneration (Quiroga et al., 2017). The non-localized maximum shear strain at each intended integration point (*ip*) was obtained as:

(14) $$\varepsilon_{\text{max},\text{nl},ip}=\frac{\sum_{intp=1}^{nip}\omega_{ip,intp}\left(\varepsilon_{\text{max},ip}\right)}{\sum_{intp=1}^{nip}\omega_{ip,intp}},$$

where *intp* and *nip* are the index and the total number of integration points in the FE model and ω_*ip*,*intp*_ is the Gauss weighting function at the intended integration point (*ip*) concerning each of other integration points (*intp*) and was obtained as:

(15) $$\begin{array}{c} \omega_{ip,intp}=\frac{1}{{\left(2\pi \right)}^{3/2}l^{3}}\\ \text{exp}\left[-\frac{\sqrt{{\left(x_{intp}-x_{ip}\right)}^{2}{\left(y_{intp}-y_{ip}\right)}^{2}{\left(z_{intp}-z_{ip}\right)}^{2}}}{2l^{2}}\right], \end{array}$$

where *x_j_*, *y_j_*, and *z_j_* are the coordinates of intended and other integration points and *l* is the characteristic length, which is a property related to the scale of the microstructure. This parameter was selected to be *l* = *d*_sup_ (superficial layer thickness = 0.12 × explant height, see Supplementary Table 1) (Quiroga et al., 2017). To make the damage progress independent of element size, the mesh was refined until the element size was smaller than the characteristic length and no mesh dependency was observed with more mesh refinement.

The obtained non-localized maximum shear strain at the integration points (ε_max,nl,*ip*_) was averaged over the element and the non-localized maximum shear strain at each element was obtained (ε_max,nl,*el*_). ε_max,nl,*el*_ was then used to define the relative change in the PG content in each element as (Mononen et al., 2018; Eskelinen et al., 2019):

(16) $$\begin{array}{c} PG_{el\_i+1}\\ =\left\{ \begin{array}{c} PG_{el\_i}\left(1-\frac{1}{3}\sqrt{\varepsilon_{\text{max},nl,el\_i}-K_{0\_\mathrm{PG}}}\right)if\varepsilon_{\max,\mathrm{nl},el\_i}>K_{0\_\mathrm{PG}}\\ PG_{el\_i}\;if\varepsilon_{\max,\mathrm{nl},el\_i}\leq K_{0\_\mathrm{PG}} \end{array}\right., \end{array}$$

where *i* is the number of the current iteration and *PG*_*el*_ is the relative PG content at each element with *PG*_*el*_0_ = 1. The higher the ε_max,nl,*el*_*i*_, the faster the PG content coefficient decreases *via* consecutive loading iterations of arbitrary time (Eskelinen et al., 2019).

The relative PG content was used to linearly modulate the FCD content as:

(17) $$FCD_{el\_i+1}=FCD_{el,0}PG_{el\_i+1},$$

where *FCD*_*el*,0_ is the initial FCD content at the element (see Supplementary Table 1). Subsequently, the relative PG content was also used to modulate tissue hydration as:

(18) $$n_{\text{f},el\_i+1}=1+PG_{el\_i+1}\left(1-n_{\text{f},el\_0}\right),$$

where *n*_f,*el*_0_ is the initial fluid volume fraction at the element (see Supplementary Table 1).

### Characterization of Degeneration Effect on the Overall Mechanical Response of the Cartilage Explants

To evaluate the combined effect of the above-described degenerative changes on the overall mechanical response of cartilage explants, the equilibrium modulus before and after degeneration was characterized for all explant models. To this end, a stress-relaxation test was simulated: after a free swelling step, a 10% compressive strain at 10%/s was applied to the top surface followed by a 60 min relaxation. This simulation was repeated for the different explants in each of FE model groups A (reference model), B (injurious loading model), and C (focal defect models) in Figure 2 presenting the initial FCD, water and collagen contents and initial fibril orientation as well as the contents and orientation obtained after the adaptive degeneration modeling (detailed above). The equilibrium modulus of the explant was obtained by dividing the equilibrium reaction stress at the explant bottom surface with the applied strain on the top surface.

## Results

### CARED Model Results

#### Reference Model (Normal Loading of the Intact Explant Model)

**FIGURE 3:**
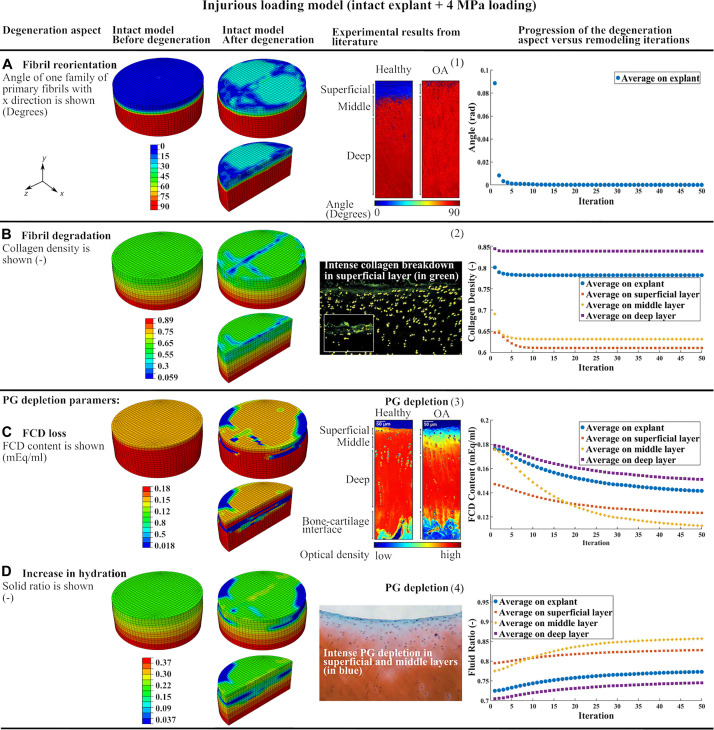
The results of the intact explant model subjected to injurious loading showing the degeneration of main constituents and comparison to previous experimental results: **(A)** fibril reorientation compared to (1) (Makela et al., 2012; Mononen et al., 2018), **(B)** fibril degradation compared to (2) (Lin et al., 2004), **(C,D)** the FCD loss and increase in tissue hydration compared to (3) (Mononen et al., 2018), and (4) (Lin et al., 2004). The curves of progression of the degeneration aspects vs. remodeling iterations in each of the three cartilage layers and over the whole tissue are shown.

The reference CARED model under normal loading (Figure 2) showed negligible fibril reorientation and degradation, FCD loss and change in tissue hydration. The obtained results were similar to the shown constituents for the intact model before degeneration in Figure 3.

#### Injurious Loading of the Intact Explant Model

Curves of degeneration progression vs. remodeling iterations (Figure 3) show the progression of the degeneration in each constituent (Eskelinen et al., 2019) in every cartilage layer and bulk tissue. The degeneration in each constituent reached a stable value after 50 iterations. The FCD loss and increase in tissue hydration had slower convergence compared to collagen fibril-related parameters (fibril reorientation and change in fibril density) which converged after 10 iterations.

Injurious loading (4 MPa compression in 0.1 s) caused horizontal fibrils in the superficial layer to reorient up to 30 degrees toward perpendicular direction of the explant surface (Figure 3A). The maximum degree of collagen fibril degradation occurred in the superficial and middle zones. More intense fibril degradation was observed in the superficial zone and in the direction perpendicular to the initial fibril orientation (compare the blue zone in Figure 3B and fibril orientation in the superficial zone in Figure 1A). The FCD loss and increase in tissue hydration initiated from the superficial layer and propagated to the middle and deep layers (Figures 3C,D). After 50 iterations, the largest FCD loss and increase in tissue hydration occurred in the middle layer. As a result, the equilibrium modulus of the explant was reduced by 33% after the simulated degeneration (Table 1).

**TABLE 1:** Equilibrium moduli of the simulated explants before and after degeneration obtained from *in silico* stress-relaxation tests.

Injury models		Initial equilibrium modulus (MPa)	Equilibrium modulus after degeneration (MPa)	Change in equilibrium modulus (%)
Injurious loading in the intact explant model		1.62	1.08	−33
Normal loading in the focal defect models	ICRS grade 1 defect	1.61	1.60	−1
	ICRS grade 2 defect	1.60	1.57	−2
	ICRS grade 3 defect	1.58	1.41	−11

#### Normal Loading of the Focal Defect Models

**FIGURE 4:**
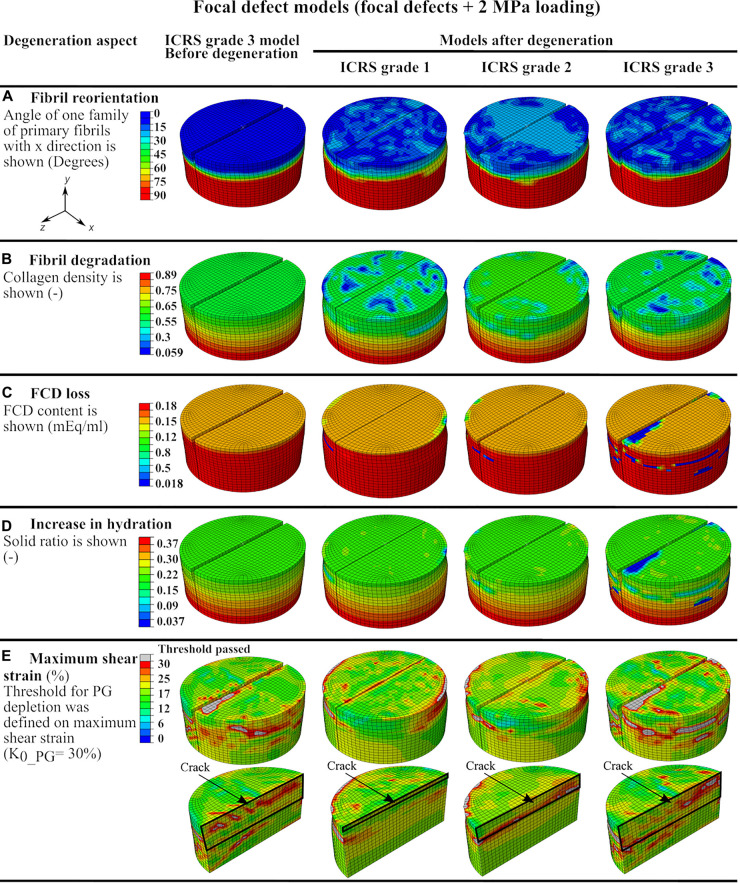
The defect model results showing color maps of degeneration aspects of the CARED model before degeneration (only for ICRS grade 3 model) and after degeneration (for the three ICRS grades models): **(A)** fibril reorientation, **(B)** fibril degradation, **(C,D)** the FCD loss and the increase in the tissue hydration and **(E)** the maximum shear strain.

The explant models with focal defects mimicking lesions of ICRS grade 1, 2, and 3 experienced moderate fibril reorientation around the defects in the superficial and middle zones and at the interface between the middle and deep zones (Figure 4A). The reorientation in the superficial zone of the ICRS grade 2 model occurred over a larger homogeneous area. On the other hand, more intense, non-homogeneous fibril degradation was observed in the superficial layers of ICRS grades 1 and 3 models (Figure 4B). The FCD loss and increase in tissue hydration (coupled to PG depletion) around the crack were more pronounced in the ICRS grade 3 model (Figures 4C,D). Tissue degeneration decreased the equilibrium modulus by 1, 2, and 11% in the ICRS grade 1, 2, and 3 models, respectively. The maximum shear strain (the parameter used as a threshold for PG depletion in CARED model) was higher around the bottom of the lesion, where strain concentration occurred, and the area of high maximum shear strain increased as a function of tissue depth for deeper lesions (Figure 4E).

## Discussion and Conclusion

The proposed CARED model in this paper provides a unique tool to overcome earlier limitations of cartilage degeneration algorithms allowing the elucidation of the contribution of different cartilage constituents to the onset and progression of OA. This was made possible through the following novel improvements:

1. Integrating the different aspects of articular cartilage degeneration (fibril reorientation and degradation and PG depletion) in a unique FE framework. Specifically integrating the fibril reorientation mechanism with other degradation mechanisms that allows studying interactive effects.
2. Using an adaptation algorithm to change the contents of cartilage constituents (collagen, FCD, and water contents) instead of changing the material properties, as implemented in earlier studies (Wilson et al., 2006a, b; Liukkonen et al., 2017; Quiroga et al., 2017; Mononen et al., 2018; Tanska et al., 2018).
3. Integrating collagen degradation by adapting a collagen degradation theory from arterial tissue (Valentín et al., 2013; Famaey et al., 2018) to cartilage.
4. Using a non-localization theory for PG depletion.
5. Coupling PG depletion, FCD loss and increase in tissue hydration.
6. Introducing a novel algorithm for the increase in tissue hydration. Variations in tissue hydration is an important parameter in fibril disorganization and may play a central role in the progression of OA (Sah et al., 1991). To the author’s knowledge, the CARED model is the first cartilage adaptive model to include variations in tissue hydration. The CARED model was integrated into a validated FRPVES material model of cartilage to predict the degeneration in intact cartilage explants subjected to normal and injurious loading and explants with defects corresponding to ICRS grades 1, 2, and 3 subjected to normal loading. In the following, the CARED model results for the different applications are discussed in comparison to earlier experimental observations.

### CARED Model Qualitative Validation in Comparison With Experiments and Other Modeling Approaches

#### Reference Model (Normal Loading of the Intact Explant Model)

As expected, the implementation of CARED model on the FE simulation of the intact cartilage explant under normal loading (reference model in Figure 2) caused minimal degenerative changes in the contents of main constituents of the explant (i.e., less than five degrees change in fibril orientation and almost no change in fibril and PG contents). Similarly, minimal or no variations in the fibril orientation and FCD content were observed in other computational studies, where either reorientation of fibrils or FCD loss of intact explant under normal loading was adaptively simulated (Tanska et al., 2018; Eskelinen et al., 2019).

#### Injurious Loading of the Intact Explant Model

The CARED model can predict collagen fibril reorientation and its protective role during injurious loading. Indeed, this study simulates the fibril reorientation in a cartilage explant subjected to injurious loading which caused horizontal fibrils in the superficial layer to reorient toward the perpendicular direction of the explant surface (Figure 3A). This is in agreement with experimental results that confirm the tendency of the fibrils to reorient toward the perpendicular direction of the cartilage surface in OA cartilage (Makela et al., 2012) (Figure 3A). Based on our model results, this reorientation can be interpreted as a protective behavior to avoid fibrils degradation. This is a speculation based on the model results and should be verified with experiments. Indeed, during cartilage compression, the tissue expands in the horizontal direction and tensile strain is applied to horizontally oriented fibrils in the superficial layer. In the case of injurious compression, this tensile strain may pass the fibrils strength threshold and causes fibril degradation. The predicted reorientation of fibrils toward the perpendicular direction of the explant surface during injurious compression caused less tensile strain to be applied to the fibrils and therefore protect fibril degeneration.

The intense collagen degradation predicted in the superficial layer of the injurious loading model (Figure 3B) is in agreement with earlier experimental and computational observations. Earlier experiments show that 5 MPa compression loading of bovine cartilage explants for 24 h caused intense collagen breakdown in the superficial layer (Lin et al., 2004) (Figure 3B). Interestingly, the intense fibril degradation in the surface of our model propagated perpendicular to the initial splitline direction (compare the blue zone in Figure 3B and initial fibril orientation in the superficial zone in Figure 1A), where the tissue has less tensile stiffness.

In the CARED model, FCD loss and increase in tissue hydration as consequences of PG depletion started from the superficial layer and propagated to the middle and deep layers with a sharp transition between PG-rich and PG-poor tissue (Figures 3C,D). This is in agreement with experimental studies that suggest PG depletion begins at the articular surface. Comparable to our simulation results, experimental data confirmed that the PG depleted area increased with increased loading, however, a sharp transition remains between the PG-rich and the PG-poor tissue (Lin et al., 2004; Mononen et al., 2018; Figures 3C,D). Comparable to our model results (Figure 3D), other experimental studies indicate that high amplitude static or cyclic loadings increase overall tissue hydration (Sah et al., 1991). In addition, our model provides the opportunity to study factors affecting the local increases in tissue hydration, which is challenging experimentally. One should note that the changes in tissue hydration shown in Figure 3D is the cumulative effect of increase in hydration as a result of solid PG loss and decrease in water content as a result of FCD loss. However, since the loading was relatively fast (0.1 s) and decrease in water content due to FCD loss is a time-dependent behavior, an overall increase in tissue hydration was predicted by the model.

In agreement with the CARED model results, earlier experimental observations suggest that an injurious mechanical loading changes the orientation of fibrils from parallel to the surface toward perpendicular to the surface followed by fibrillation and collagen fibril degradation, especially at the cartilage surface (Makela et al., 2012; Goldring and Goldring, 2016). In our model, the fibrillation mechanism is indirectly covered with including the fibril degradation algorithm. Since the change in orientation of the fibrils in the model is a consequence of the high rate traumatic loading (4 MPa compression in 0.1 s), it simulates an instantaneous reorientation. This instantaneous change in fibrils orientation affected the strain field in the cartilage and consequently the fibril degradation, which was suggested by experimental studies (Karsdal et al., 2008; Bay-Jensen et al., 2010) to be an irreversible permanent phenomena due to the long half-life of collagen fibrils (Verzijl et al., 2000). Furthermore, in early OA and especially in the superficial layer, high water content, elevated strains, disorganized collagen fibrils and decreased FCD content are found (Wong et al., 2008; Saarakkala et al., 2010). The proposed adaptive model by Hosseini et al. (2014) predicted more ground substance softening over a larger area than collagen damage. Comparing Figures 3B,C, a similar conclusion can be made based on the results of the CARED model. According to our model results, the maximum reduction in PG and fibril contents occurred in the middle layer (compare differences between initial and final iterations in curves of different layers in Figures 3B–D) in agreement with recent experimental results (Durney et al., 2020). In the CARED model, the equilibrium modulus of the simulated explant was reduced by 33% following the degeneration (Table 1). Time-dependent reduction in mechanical properties with the progression of cartilage damage is known to occur experimentally and clinically (Kempson et al., 1973; Buckwalter et al., 1994; McCormack and Mansour, 1998; Hosseini et al., 2014).

The predicted fibril and PG degenerations in this study are closer to experimental results compared to earlier degeneration modeling approaches. In the model proposed by Hosseini et al. (2014) the fibril and PG degenerations only occurred locally and around the point of injurious indentation loading, while our model predicted variable degradation levels in the superficial, middle and deep zones of the explant (curves in Figures 3B–D). This is in agreement with experimentally observed fibril and PG degenerations in cartilage explants subjected to injurious loading (Lin et al., 2004) (see Figures 3B,D: green points showing fibril degradation and blue zones showing PG depletion in different layers). This difference in model predictions may be related to the 2D geometry, local indentation loading, different implementation of degeneration algorithm (changing material properties instead of cartilage constituents), using a local PG depletion algorithm (in comparison with the non-local algorithm used in CARED model) and neglecting the fibril reorientation effect in the model proposed by Hosseini et al. (2014). Similar geometry, material model and degeneration algorithm to our model were used by Eskelinen et al. (2019) to predict FCD loss in a cartilage explant under different loading conditions. However, in their model under injurious loading (4 MPa compression in 0.1 s, comparable to the current study) FCD loss was only detected at the edges of the superficial layer of the explant. This is in contrast with the experimental results suggesting more homogeneous PG depletion over the superficial and middle layers, with a higher concentration in the superficial edges (Lin et al., 2004). This difference can be explained by neglecting the degenerative changes related to fibrils degradation and disorganization and an increase in tissue hydration. Integrating all these degenerative changes in the CARED model resulted in more consistent prediction of FCD loss location with experiments in comparison with the earlier adaptive degeneration models that do not account for one or more of the main cartilage degenerative changes included in the CARED model (see color map in Figure 3C). In particular, the CARED model predicted FCD loss in the middle of the superficial layer of the explant (indicated in green color in Figure 3C) and more concentrated FCD loss in the edges and middle layer (indicated in blue color in Figure 3C), which was also observed experimentally (Lin et al., 2004).

#### Normal Loading of the Focal Defect Models

The integration of both fibril reorientation and degradation algorithms in the CARED model allowed the investigation of the interaction between fibril reorientation and degradation by comparing the relative results (Figures 4A,B). All models of explants with ICRS grades 1, 2, and 3 experienced fibril reorientation around the defects in the superficial, middle and at the interface between middle and deep layers (Figure 4A). This is in agreement with previous experimental results showing the fibrillation of the collagen fibril network to occur near the experimentally produced partial-thickness defects (Lyman et al., 2012). Similarly, in the fibril reorientation model proposed by Tanska et al. (2018) disorganization of collagen fibrils was observed around the focal defects in the modeled explant (Tanska et al., 2018) presumed that this breakdown of the collagen fibrils could be the reason for the reorientation of fibrils. However, the model with ICRS grade 2 defect presented maximum fibril reorientation over a large area in the explant surface and around the crack, with only minimal fibril degradation at the same location of fibril reorientation. This suggests that fibril reorientation may prevent or slow down fibril degradation.

Cartilage lesion depth is a crucial parameter affecting the fibril degradation behavior. The model with ICRS grade 1 defect experienced more intense fibril degradation in the superficial layer than the models with deeper defects (ICRS grades 2 and 3 in Figure 4B). This can be explained by the fact that in the ICRS grade 1 model, the bottom of the crack, where strain concentration occurs (see strain results of grade 1 model in Figure 4E), was located in the superficial layer with less collagen density and fibrils oriented parallel to the surface and normal to the crack direction. This caused a higher strain in the direction of fibrils in a zone with minimal fibril density thereby increasing fibril degradation. The fibril degradation in the model with the ICRS grade 3 defect exceeded fibril degradation in the ICRS grade 2 model. Although in both models the cracks were outside the superficial zone, the deeper crack in the ICRS grade 3 model caused more deformation in the superficial zone under compressive loading. This increased the strain applied to the fibrils in the superficial zone with minimum fibrils density and resulted in more fibril degradation in the ICRS grade 3 model.

Maximum PG depletion in all the focal defect models was observed around the cracks as reflected by the FCD loss and increase in hydration in Figures 4C,D. These predictions are similar to the experimental observations suggest concentration of FCD loss around cartilage defects (Orozco et al., 2018). The maximum shear strain fields in the models that determine PG depletion (Figure 4E) show that the PG depletion threshold, here set at maximum shear strain = 30%, was passed around the crack bottom and opening in the superficial zone. More deformation caused by the compressive load in the model with ICRS grade 3 defect in comparison with grades 1 and 2 cracks caused maximum FCD loss and increase in tissue hydration in this model, which decreased the equilibrium modulus of this model more than the others (Table 1). This shows that the explant equilibrium stiffness is more dependent on PG content (FCD and fluid contents in the model) than collagen content and organization.

### Predicted Interaction Between Collagen and PG Degeneration by the CRAED Model

The degeneration rates in CARED model (fibril reorientation rate in Equation 7, fibril degradation rate in Equation 12 and PG depletion rate in Equation 16 were selected in agreement with previous computational studies and based on experimental observations (Valentín et al., 2013; Famaey et al., 2018; Tanska et al., 2018; Eskelinen et al., 2019). These degeneration rates present the changes in the contents of cartilage constituents over an arbitrary time. To accurately validate the model, the degeneration rates need to be further calibrated based on *in vitro* or *in vivo* experiments. To this end, the specific experimentally applied cartilage loading must be replicated in the FE model. In the absence of this information, in our implementation, each iteration in the CARED model reflects an arbitrary time step of cartilage loading until response convergence. Given the very simple loading conditions (compressive pressure of 2 or 4 MPa in 0.1 s) this is acceptable, however, this will cause the model results to most likely reflect the extreme course of degeneration as there is no constituent recovery due to intermittent relative unloading.

Experimental observations suggest the presence of interaction between PG and collagen degradations in cartilage (Inamdar et al., 2019). The CARED model explicitly integrates the degenerative changes in the contents of collagen and PG constituents and therefore allows the elucidation of the interaction between the degenerative changes in the contents of individual constituents. To this end, the curves of degeneration aspects vs. iterations under injurious loading (Figure 3) can be used. These curves show that most of the degenerative variations in the collagen fibril network (fibril reorientation and degradation) occurred in the first iteration, while the variations in the PG related contents (FCD and water contents) occurred over the progression of 50 iterations with a relatively slower rate in the first iteration than between subsequent iterations. This shows that during the first time iteration of the specific applied injurious loading, collagen fibril reorientation and degradation are occurring at higher rates than PG depletion. Then, the fibril variations increased the FCD loss and tissue hydration in the next iterations, which shows the amplifying effect of fibril degradation on PG depletion. However, following these first iterations, despite the continued decrease in FCD content and increase in water content (Figures 3C,D), no additional change in collagen content and orientation was observed (Figures 3A,B). Fibril degradation induced by the increased strain in fibrils direction in the first iteration due to the fast injurious loading (4 MPa in 0.1 s), may explain this effect. Indeed, the initial decrease in fibril density caused more pronounced tissue deformation after the first iteration and increased the maximum shear strain in the tissue, thereby inducing even more FCD loss and an increase in tissue hydration. In other words, as the fibrils support the tensile load in their direction during pressurization, the initial increase in fibril degradation accelerates PG depletion.

### Limitations

The current degeneration model has been validated comparable to other modeling approaches in the literature and experimental results. Although our results present good agreement with experimental results and sometimes better agreement compared to previous modeling approaches, several knowledge voids exist. The proposed values for the defined degeneration thresholds are largely variable in the literature. Here we used thresholds that are in agreement with the earlier experimental observations and are proven to affect cartilage tissue degeneration. Accurate validation of the model requires a set of experiments to characterize the material, structural and compositional properties of cartilage explants and determine the thresholds for each of the PG and collagen degeneration algorithms. Another set of experiments would be required to validate the CARED model by measuring the fibril reorientation and degradation as well as PG depletion in cartilage explants after going through similar loading conditions as applied to the model. Previously reported sensitivity analysis of the damage threshold values (Hosseini et al., 2014; Eskelinen et al., 2019) shows their effect in terms of the size of the affected area and the severity of the damage. However, damage location, time-dependent damage progression patterns and the nature of the interaction between damage in the PG and the collagen fibrils are insensitive to these parameters. Therefore, the results of the CARED model with the current threshold values can be used to further elucidate the degenerative behavior of the cartilage tissue under mechanical loading. Furthermore, the purpose of this research was not to mimic the individual degeneration of a cartilage explant and validate the predictions but to merely proposing a model which can be used to look at the interaction between different degeneration mechanisms.

Unconfined compression was used as the loading configuration in our FE models, since it is often used in *in vitro* experimental studies (Gratz et al., 2009; Szarko and Xia, 2012; Li et al., 2013) due to its easy experimental setup. Therefore, more validation experiments are available in literature than other loading setups. Yet, we acknowledge that the loading in unconfined geometry can be considered as an idealization and limitation, and *in vivo* loading on cartilage is more complex. In future, other types of loading conditions (e.g., confined compression, indentation or physiological joint loading) should be used to validate the proposed model.

Finally, although the applied loadings on the explant FE models were justified by *in vitro* literature, they do not simulate precise *in vivo* normal or injurious loading for the different explants from various species or patients. More accurate definitions for normal and injurious loadings have to be determined using *in vivo* experimental results. Similarly, the rates of fibril reorientation and degradation and PG depletion (Equations 7, 12, 16) need to be optimized to reflect a physiological timescale according to experimental results, instead of arbitrary time scale. By scaling one iteration step to correspond to the fibril degradation and PG depletion in one loading step as observed in an *in vitro* experiment, this would enhance the *in vivo* use as the degeneration rate could then be scaled, for example, to the degeneration in half a year of walking (Eskelinen et al., 2019).

In conclusion, CARED model proposed an *in silico* integrated framework to predict the cartilage degeneration through changes in the contents of its constituents. This framework includes the degenerative changes in collagen fibril content (adapted from arterial tissue to cartilage) and orientation (implemented from a previous study), FCD and water contents (increase in water content was introduced for the first time and together with a previously developed FCD loss model were linked to PG depletion). Our model allows the observation of local degenerative changes in 3D geometry of cartilage, which is challenging in *in vitro* and *in vivo* experiments in particular for local increase in tissue hydration. The reorientation and degeneration algorithms implemented in the CARED model show a good agreement with experiments reported in the literature in terms of the trend and location of changes within the tissue following injurious loading and presence of defects. In addition, the proposed integrated model enabled the study of the interaction between the degenerative changes in the contents of cartilage constituents following injurious loading of intact cartilage tissue as well as physiologic loading of defect cartilage. The model confirms the role of fibrils degradation as a key parameter in the irreversible progression of cartilage degeneration and OA, as it was suggested by previous studies (Karsdal et al., 2008; Bay-Jensen et al., 2010; Tanska et al., 2018). Using the CARED model, different aspects of cartilage tissue degeneration under different mechanical conditions (e.g., under injurious compressive or shear loadings) or in the presence of various defects can be studied. As a next step, the model will be used together with a whole knee joint FE simulation to study the cartilage degeneration in comparison with *in vivo* longitudinal experiments.

## Data Availability Statement

The raw data supporting the conclusions of this article will be made available by the authors, without undue reservation.

## Author Contributions

SE: conceptualization, methodology, software, validation, formal analysis, investigation, data curation, writing – original draft, writing – review and editing, visualization, and funding acquisition. PT: conceptualization, methodology, software, and writing – review and editing. RK: conceptualization, methodology, writing – review and editing, and supervision. RL: conceptualization, supervision, and funding acquisition. NF: conceptualization, methodology, resources, writing – review and editing, funding acquisition, and supervision. IJ: conceptualization, methodology, resources, writing – review and editing, funding acquisition, and supervision. All authors contributed to the article and approved the submitted version.

## Conflict of Interest

The authors declare that the research was conducted in the absence of any commercial or financial relationships that could be construed as a potential conflict of interest. The handling editor declared a past co-authorship with one of the author NF.

## References

[B1] Bay-Jensen A. C. Hoegh-Madsen S. Dam E. Henriksen K. Sondergaard B. C. Pastoureau P. (2010). Which elements are involved in reversible and irreversible cartilage degradation in osteoarthritis? *Rheumatol. Int.* 30 435–442. 10.1007/s00296-009-1183-1 19816688

[B2] Buckwalter J. A. (1992). Mechanical injuries of articular cartilage. *Iowa Orthop. J.* 12 50–57.

[B3] Buckwalter J. A. Mow V. C. Ratcliffe A. (1994). Restoration of injured or degenerated articular cartilage. *J. Am. Acad. Orthop. Surg.* 2 192–201. 10.5435/00124635-199407000-00002 10709009

[B4] Clark J. M. (1985). The organization of collagen in cryofractured rabbit articular cartilage: a scanning electron microscopic study. *J. Orthop. Res.* 3 17–29. 10.1002/jor.1100030102 3981292

[B5] Da Silva M. A. Yamada N. Clarke N. M. Roach H. I. (2009). Cellular and epigenetic features of a young healthy and a young osteoarthritic cartilage compared with aged control and OA cartilage. *J. Orthop. Res.* 27 593–601. 10.1002/jor.20799 18985702

[B6] Driessen N. J. B. Boerboom R. A. Huyghe J. M. Bouten C. V. C. Baaijens F. P. T. (2003). Computational analyses of mechanically induced collagen fiber remodeling in the aortic heart valve. *J. Biomech. Eng.* 125 549–557. 12968580 10.1115/1.1590361

[B7] Driessen N. J. B. Bouten C. V. C. Baaijens F. P. T. (2005). Improved prediction of the collagen fiber architecture in the aortic heart valve. *J. Biomech. Eng.* 127 329–336. 10.1115/1.1865187 15971711

[B8] Dulay G. S. Cooper C. Dennison E. M. (2015). Knee pain, knee injury, knee osteoarthritis & work. *Best Pract. Res. Clin. Rheumatol.* 29 454–461. 10.1016/j.berh.2015.05.005 26612241

[B9] Durney K. M. Shaeffer C. A. Zimmerman B. K. Nims R. J. Oungoulian S. Jones B. K. (2020). Immature bovine cartilage wear by fatigue failure and delamination. *J. Biomech.* 23:109852. 10.1016/j.jbiomech.2020.10985 PMC7809915 32517855

[B10] Eskelinen A. S. A. Mononen M. E. Venäläinen M. S. Korhonen R. K. Tanska P. (2019). Maximum shear strain-based algorithm can predict proteoglycan loss in damaged articular cartilage. *Biomech. Model. Mechanobiol.* 18 753–778. 10.1007/s10237-018-01113-1 30631999

[B11] Famaey N. Vastmans J. Fehervary H. Maes L. Vanderveken E. Rega F. (2018). Numerical simulation of arterial remodeling in pulmonary autografts. *J. Appl. Math. Mech.* 98 2239–2257. 10.1002/zamm.201700351

[B12] Ferizi U. Rossi I. Lee Y. Lendhey M. Teplensky J. Kennedy O. D. (2017). Diffusion tensor imaging of articular cartilage at 3T correlates with histology and biomechanics in a mechanical injury model. *Mag. Reson. Med.* 78 69–78. 10.1002/mrm.26336 27455389 PMC9175493

[B13] Fox A. J. S. Bedi A. Rodeo S. A. (2009). The basic science of articular cartilage: structure, composition, and function. *Sports Health* 1 461–468. 10.1177/1941738109350438 23015907 PMC3445147

[B14] Gardiner B. S. Woodhouse F. G. Besier T. F. Grodzinsky A. J. Lloyd D. G. Zhang L. (2016). Predicting knee osteoarthritis. *Ann. Biomed. Eng.* 44 222–233. 10.1007/s10439-015-1393-5 26206679 PMC4690844

[B15] Goldring S. R. Goldring M. B. (2016). Changes in the osteochondral unit during osteoarthritis: structure, function and cartilage–bone crosstalk. *Nat. Rev. Rheumatol.* 12:632. 10.1038/nrrheum.2016.148 27652499

[B16] Gratz K. Wong B. Bae W. Sah R. (2009). The effects of focal articular defects on cartilage contact mechanics. *J. Orthop. Res.* 27 584–592. 10.1002/jor.20762 18979528 PMC2862585

[B17] Horton W. E. Bennion P. Yang L. (2006). Cellular, molecular, and matrix changes in cartilage during aging and osteoarthritis. *J. Musculoskelet. Neuronal Interact.* 6 379–381. 17185833

[B18] Hosseini S. M. Wilson W. Ito K. van Donkelaar C. C. (2014). A numerical model to study mechanically induced initiation and progression of damage in articular cartilage. *Osteoarthr. Cartil.* 22 95–103. 10.1016/j.joca.2013.10.010 24185112

[B19] Inamdar S. R. Barbieri E. Terrill N. J. Knight M. M. Gupta H. S. (2019). Proteoglycan degradation mimics static compression by altering the natural gradients in fibrillar organisation in cartilage. *Acta Biomater.* 97 437–450. 10.1016/j.actbio.2019.07.055 31374336 PMC6838783

[B20] Julkunen P. Wilson W. Isaksson H. Jurvelin J. S. Herzog W. Korhonen R. K. (2013). A review of the combination of experimental measurements and fibril-reinforced modeling for investigation of articular cartilage and chondrocyte response to loading. *Comput. Math. Methods Med.* 2013:326150. 10.1155/2013/326150 23653665 PMC3638701

[B21] Karsdal M. A. Madsen S. H. Christiansen C. Henriksen K. Fosang A. J. Sondergaard B. C. (2008). Cartilage degradation is fully reversible in the presence of aggrecanase but not matrix metalloproteinase activity. *Arthritis Res. Ther.* 10:R63. 10.1186/ar2434 PMC2483454 18513402

[B22] Keenan K. E. Pal S. Lindsey D. P. Besier T. F. Beaupre G. S. (2013). A viscoelastic constitutive model can accurately represent entire creep indentation tests of human patella cartilage. *J. Appl. Biomech.* 29 292–302. 10.1123/jab.29.3.292 23027200 PMC3896388

[B23] Kempson G. E. Muir H. Pollard C. Tuke M. (1973). The tensile properties of the cartilage of human femoral condyles related to the content of collagen and glycosaminoglycans. *Biochem. Biophys. Acta* 297 465–472. 10.1016/0304-4165(73)90093-7 4267503

[B24] Kłodowski A. Mononen M. E. Kulmala J. P. Valkeapää A. Korhonen R. K. Janne A. (2016). Merge of motion analysis, multibody dynamics and finite element method for the subject-specific analysis of cartilage loading patterns during gait: differences between rotation and moment-driven models of human knee joint. *Multibody Syst. Dyn.* 37 271–290. 10.1007/s11044-015-9470-y

[B25] Koh Y. G. Lee J. A. Kim Y. S. Lee H. Y. Kim H. J. Kang K. T. (2019). Optimal mechanical properties of a scaffold for cartilage regeneration using finite element analysis. *J. Tissue Eng.* 10:2041731419832133. 10.1177/2041731419832133 PMC6396049 30834102

[B26] Li Y. Frank E. Wang Y. Chubinskaya S. Huang H. Grodzinsky A. (2013). Moderate dynamic compression inhibits pro-catabolic response of cartilage to mechanical injury, TNF-α and IL-6, but accentuates degradation above a strain threshold. *Osteoarthr. Cartil.* 21 1933–1941. 10.1016/j.joca.2013.08.021 24007885 PMC3855909

[B27] Lin P. M. Chen C. T. Torzilli P. A. (2004). Increased stromelysin-1 (MMP-3), proteoglycan degradation (3B3-and 7D4) and collagen damage in cyclically load-injured articular cartilage. *Osteoarthr. Cartil.* 1 485–496. 10.1016/j.joca.2004.02.012 15135145

[B28] Liukkonen M. K. Mononen M. E. Klets O. Arokoski J. P. Saarakkala S. Korhonen R. K. (2017). Simulation of subject-specific progression of knee osteoarthritis and comparison to experimental follow-up data: data from the osteoarthritis initiative. *Sci. Rep.* 7:9177. 10.1038/s41598-017-09013-7 28835668 PMC5569023

[B29] Loening A. M. James I. E. Levenston M. E. Badger A. M. Frank E. H. Nuttall M. E. (2000). Injurious mechanical compression of bovine articular cartilage induces chondrocyte apoptosis. *Arch. Biochem. Biophys.* 381 205–212. 10.1006/abbi.2000.1988 11032407

[B30] Loening A. M. Levenston M. E. James I. E. Nuttall M. E. Gowen M. Grodzinsky A. J. (1999). “Injurious compression of bovine articular cartilage induces chondrocyte apoptosis before detectable mechanical damage,” in *Proceedings of the 45th Annual Meeting, Orthopaedic Research Society*, Anaheim, CA, 42.

[B31] Lyman J. R. Chappell J. D. Morales T. I. Kelley S. S. Lee G. M. (2012). Response of chondrocytes to local mechanical injury in an ex vivo model. *Cartilage* 3 58–69. 10.1177/1947603511421155 26069619 PMC4297183

[B32] Makela J. T. A. Huttu M. R. J. Korhonen R. K. (2012). Structure-function relationships in osteoarthritic human hip joint articular cartilage. *Osteoarthr. Cartil.* 20 1268–1277. 10.1016/j.joca.2012.07.016 22858669 PMC3627049

[B33] McCann L. Ingham E. Jin Z. Fisher J. (2009). Influence of the meniscus on friction and degradation of cartilage in the natural knee joint | elsevier enhanced reader. *Osteoarthr. Cartil.* 17 995–1000. 10.1016/j.joca.2009.02.012 19328878

[B34] McCormack T. Mansour J. M. (1998). Reduction in tensile strength of cartilage precedes surface damage under repeated compressive loading in vitro. *J. Biomech.* 31 55–61. 10.1016/s0021-9290(97)00103-6 9596538

[B35] Men Y.-T. Li X.-M. Chen L. Fu H. (2017). Experimental study on the mechanical properties of porcine cartilage with microdefect under rolling load. *J. Healthc. Eng.* 2017:2306160. 10.1155/2017/2306160 PMC5485335 29065577

[B36] Mohammadi H. Mequanint K. Herzog W. (2013). Computational aspects in mechanical modeling of the articular cartilage tissue. *Proc. Inst. Mech. Eng. H* 227 402–420. 10.1177/0954411912470239 23637216

[B37] Mononen M. E. Mikkola M. T. Julkunen P. Ojala R. Nieminen M. T. Jurvelin J. S. (2012). Effect of superficial collagen patterns and fibrillation of femoral articular cartilage on knee joint mechanics-a 3D finite element analysis. *J. Biomech.* 45 579–587. 10.1016/j.jbiomech.2011.11.003 22137088

[B38] Mononen M. E. Tanska P. Isaksson H. Korhonen R. K. (2016). A novel method to simulate the progression of collagen degeneration of cartilage in the knee: data from the osteoarthritis initiative. *Sci. Rep.* 6:21415. 10.1038/srep21415 26906749 PMC4764929

[B39] Mononen M. E. Tanska P. Isaksson H. Korhonen R. K. (2018). New algorithm for simulation of proteoglycan loss and collagen degeneration in the knee joint: data from the osteoarthritis initiative. *J. Orthop. Res.* 36 1673–1683. 10.1002/jor.23811 29150953

[B40] Mukherjee S. Nazemi M. Jonkers I. Geris L. (2020). Use of computational modeling to study joint degeneration: a review. *Front. Bioeng. Biotechnol.* 8:93. 10.3389/fbioe.2020.00093 32185167 PMC7058554

[B41] Nagel T. Kelly D. J. (2013). The composition of engineered cartilage at the time of implantation determines the likelihood of regenerating tissue with a normal collagen architecture. *Tissue Eng. Part A* 19 824–833. 10.1089/ten.tea.2012.0363 23082998

[B42] Orozco G. A. Bolcos P. Mohammadi A. Tanaka M. S. Yang M. Link T. M. (2020). Prediction of local fixed charge density loss in cartilage following ACL injury and reconstruction: a computational proof-of-concept study with MRI follow-up ACL reconstruction. *J. Orthop. Res* 39 1064–1081. 10.1002/jor.24797 32639603 PMC7790898

[B43] Orozco G. A. Tanska P. Florea C. Grodzinsky A. J. Korhonen R. K. (2018). A novel mechanobiological model can predict how physiologically relevant dynamic loading causes proteoglycan loss in mechanically injured articular cartilage. *Sci. Rep.* 8:15599. 10.1038/s41598-018-33759-3 30348953 PMC6197240

[B44] Quinn T. M. Allen R. G. Schalet B. J. Perumbuli P. Hunziker E. B. (2001). Matrix and cell injury due to sub-impact loading of adult bovine articular cartilage explants: effects of strain rate and peak stress. *J. Orthop. Res.* 19 242–249. 10.1016/S0736-0266(00)00025-5 11347697

[B45] Quiroga J. M. P. Wilson W. Ito K. van Donkelaar C. C. (2017). The effect of loading rate on the development of early damage in articular cartilage. *Biomech. Model. Mechanobiol.* 16 263–273. 10.1007/s10237-016-0815-0 27514541 PMC5285418

[B46] Roughley P. J. Lee E. R. (1994). Cartilage proteoglycans: structure and potential functions. *Microsc. Res. Tech.* 28 385–397. 10.1002/jemt.1070280505 7919526

[B47] Saarakkala S. Julkunen P. Kiviranta P. Makitalo J. Jurvelin J. S. Korhonen R. K. (2010). Depth-wise progression of osteoarthritis in human articular cartilage: investigation of composition, structure and biomechanics. *Osteoar. Cartil.* 18 73–81. 10.1016/j.joca.2009.08.003 19733642

[B48] Sah R. L. Y. Doong J. Y. H. Grodzinsky A. J. Plaas A. H. K. Sandy J. D. (1991). Effects of compression on the loss of newly synthesized proteoglycans and proteins from cartilage explants. *Arch. Biochem. Biophys.* 286 20–29. 10.1016/0003-9861(91)90004-3 1897947

[B49] Setton L. A. Elliott D. M. Mow V. C. (1999). Altered mechanics of cartilage with osteoarthritis: human osteoarthritis and an experimental model of joint degeneration | elsevier enhanced reader. *Osteoarthr. Cartil.* 7 2–14. 10.1053/joca.1998.0170 10367011

[B50] Speirs A. D. Beaulé P. E. Ferguson S. J. Frei H. (2014). Stress distribution and consolidation in cartilage constituents is influenced by cyclic loading and osteoarthritic degeneration. *J. Biomech.* 47 2348–2353. 10.1016/j.jbiomech.2014.04.031 24856886

[B51] Szarko M. Xia Y. (2012). Direct visualisation of the depth-dependent mechanical properties of full-thickness articular cartilage. *Open. J. Orthop*. 2, 34–39. 10.4236/ojo.2012.22007 24416657 PMC3886840

[B52] Tanska P. Julkunen P. Korhonen R. K. (2018). A computational algorithm to simulate disorganization of collagen network in injured articular cartilage. *Biomech. Model. Mechanobiol.* 17 689–699. 10.1007/s10237-017-0986-3 29177932

[B53] Valentín A. Humphrey J. D. Holzapfel G. A. (2013). A finite element-based constrained mixture implementation for arterial growth, remodeling, and adaptation: theory and numerical verification. *Int. J. Numer. Method. Biomed. Eng.* 29 822–849. 10.1002/cnm.2555 23713058 PMC3735847

[B54] Verzijl N. DeGroot J. Thorpe S. R. Bank R. A. Shaw J. N. Lyons T. J. (2000). Effect of collagen turnover on the accumulation of advanced glycation end products. *J. Biol. Chem.* 275 39027–39031. 10.1074/jbc.m006700200 10976109

[B55] Wang X. Neu C. P. Pierce D. M. (2019). Advances toward multiscale computational models of cartilage mechanics and mechanobiology. *Curr. Opin. Biomed. Eng.* 11 51–57. 10.1016/j.cobme.2019.09.013

[B56] Wilson W. Driessen N. J. B. van Donkelaar C. C. Ito K. (2006a). Prediction of collagen orientation in articular cartilage by a collagen remodeling algorithm. *Osteoarthr. Cartil.* 14 1196–1202. 10.1016/j.joca.2006.05.006 16797194

[B57] Wilson W. van Burken C. van Donkelaar C. Buma P. van Rietbergen B. Huiskes R. (2006b). Causes of mechanically induced collagen damage in articular cartilage. *J. Orthop. Res.* 24 220–228. 10.1002/jor.20027 16435355

[B58] Wilson W. van Donkelaar C. C. van Rietbergen B. Huiskes R. (2005). A fibril-reinforced poroviscoelastic swelling model for articular cartilage. *J. Biomech.* 38 1195–1204. 10.1016/j.jbiomech.2004.07.003 15863103

[B59] Wong B. L. Bae W. C. Chun J. Gratz K. R. Lotz M. Sah R. L. (2008). Biomechanics of cartilage articulation: effects of lubrication and degeneration on shear deformation. *Arthritis Rheum.* 58 2065–2074. 10.1002/art.23548 18576324

[B60] Wu J. Z. Herzog W. Federico S. (2016). Finite element modeling of finite deformable, biphasic biological tissues with transversely isotropic statistically distributed fibers: toward a practical solution. *Z. Angew. Math. Phys.* 67:26. 10.1007/s00033-015-0598-7 27330228 PMC4908457

