# Supplementary material for: An in silico Framework of Cartilage Degeneration That Integrates Fibril Reorientation and Degradation Along With Altered Hydration and Fixed Charge Density Loss
Source: Front Bioeng Biotechnol. 2021 Jun 22;9:680257. doi: 10.3389/fbioe.2021.680257 (PMC8258121; doi:10.3389/fbioe.2021.680257)
Supplement: Supplementary file 1 [file Data_Sheet_1.DOCX]

Supplementary Material

# FRPVES material model

A 3D description of the fibril-reinforced poro-viscoelastic material with Donnan osmotic swelling (FRPVES) (Eskelinen et al., 2019; Wilson et al., 2005a) was used to simulate the mechanics of articular cartilage. The biphasic property of cartilage was assumed in the material model by an interaction between the fluid phase (water) and the porous solid phase. The fluid flow within the tissue was considered using Darcy’s law:

| $\boldsymbol{q}=-k\nabla p,$ | (S.1) |
| --- | --- |

where ***q*** is the fluid flow flux vector and $\nabla p$ is the pore fluid pressure gradient. *k* is the current deformation-dependent permeability of the material and obtained as (Van der Voet, 1997):

| $k=k_{0}\left( \frac{1+e}{1+e_{0}} \right)^{M},$ | (S.2) |
| --- | --- |

where $k_{0}$ describes the initial permeability, *e* and $e_{0}$ are the current and initial void ratios, respectively, and *M* is permeability void-ratio dependency constant.

The solid phase was considered as a hyperelastic swelling non-fibrilar matrix describing the PG content and a viscoelastic collagen fibril network. A hyperelastic compressible Neo-Hookean definition was used to determine the stress in the non-fibrilar compartment (Wilson et al., 2005a, 2007):

| $\boldsymbol{\sigma}_{\mathrm{nf}}=\frac{1}{2}K_{\mathrm{nf}}\left( J\boldsymbol{-}\frac{1}{J} \right)\mathbf{I}+\frac{G_{\mathrm{nf}}}{J}\left( \mathbf{F}\mathbf{F}^{T}\boldsymbol{-}J^{\frac{\boldsymbol{2}}{\boldsymbol{3}}}\mathbf{I} \right),$ | (S.3) |
| --- | --- |

where **F** is the deformation gradient tensor, *J* is the determinant of **F** and **I** is the identity tensor. $K_{\mathrm{nf}}$ and $G_{\mathrm{nf}}$ are the bulk and shear moduli, respectively and can be expressed as functions of Young’s modulus ($E_{\mathrm{nf}}$) and Poisson’s ratio ($\nu_{\mathrm{nf}}$) of non-fibrilar matrix:

| $K_{\mathrm{nf}}=\frac{E_{\mathrm{nf}}}{3\left( {1-2\nu}_{\mathrm{nf}} \right)}$ | (S.4) |
| --- | --- |

and

| $G_{\mathrm{nf}}=\frac{E_{\mathrm{nf}}}{2\left( {1+\nu}_{\mathrm{nf}} \right)} .$ | (S.5) |
| --- | --- |

The Donnan osmotic swelling pressure gradient of the non-fibrilar matrix at the equilibrium is (Huyghe et al., 2003):

| $\Delta\pi=\emptyset_{int}RT\left( \sqrt{c_{F}^{2}+4\frac{\left( \gamma_{ext}^{\pm} \right)^{2}}{\left( \gamma_{int}^{\pm} \right)^{2}}c_{ext}^{2}} \right)-2\emptyset_{ext}RTc_{ext} ,$ | (S.6) |
| --- | --- |

where *R* is the molar gas constant (8.314 J/ mol K), T is the absolute temperature (293.0 K), $c_{\mathrm{ext}}$ is the external salt concentration (0.15 M), $\emptyset_{\mathrm{ext}}$, $\emptyset_{\mathrm{int}}$, $\gamma_{\mathrm{ext}}^{\pm}$ and $\gamma_{\mathrm{int}}^{\pm}$ are external and internal osmotic coefficients and external and internal activity coefficients, respectively, and $c_{\mathrm{FCD}}$ is the current depth-dependent FCD concentration and modelled as a function of volumetric deformation:

| $c_{\mathrm{FCD}}=c_{\mathrm{FCD},0}\frac{n_{f}}{n_{f}-1+J} ,$ | (S.7) |
| --- | --- |

where $c_{\mathrm{FCD},0}$ denotes the initial depth-dependent FCD content and $n_{f}$ is the fluid volume fraction.

The chemical expansion stress was expressed as (Wilson et al., 2005a):

| $T_{c}=a_{0}c_{\mathrm{FCD}}\exp\left( -\kappa_{c}\frac{\gamma_{ext}^{\pm}}{\gamma_{int}^{\pm}}\sqrt{c^{-}\left( c^{-}+c_{\mathrm{FCD}} \right)} \right) ,$ | (S.8) |
| --- | --- |

where $a_{0}$ and $\kappa_{c}$ are the material constants (Wilson et al., 2005a) and $c^{-}$ is the mobile anion concentration.

The fibrillar network was assumed not to resist compression and to have a viscoelastic stress-strain behaviour in tension:

| $\sigma_{f}=\left\{ \begin{matrix} -\frac{\eta}{2\sqrt{\left( \sigma_{f}-E_{0}\varepsilon_{f} \right)E_{\varepsilon}}}\dot{\sigma_{f}}+E_{0}\varepsilon_{f}+\left( \eta+\frac{\eta E_{0}}{2\sqrt{\left( \sigma_{f}-E_{0}\varepsilon_{f} \right)E_{\varepsilon}}} \right)\dot{\varepsilon_{f}}, \varepsilon_{f}\geq0 \\ 0, \varepsilon_{f}<0 \end{matrix} \right.,$ | (S.9) |
| --- | --- |

where $\eta$ is the viscoelastic damping coefficient and $E_{0}$ and $E_{\varepsilon}$ are initial and strain-dependent fibril network moduli, respectively, $\dot{\sigma_{f}}$ and $\dot{\varepsilon_{f}}$ are the fibril stress and strain rates, respectively and $\varepsilon_{f}$ is the logarithmic fibril strain:

| $\varepsilon_{f}=\ln\left( \left\Vert\mathbf{F}\boldsymbol{e}_{f} \right\Vert\right),$ | (S.10) |
| --- | --- |

where $\boldsymbol{e}_{f}$ is the unit vector of fibril orientation. The collagen network consists of 4 Benninghoff arcade-shape primary fibrils and 13 randomly oriented secondary fibrils. Experimental observations show that primary fibrils split lines at the superficial layer are oriented in two directions in most parts of the articular cartilage tissue (Mononen et al., 2012; Clark, 1985; Meachim et al. 1974). Therefore, the 4 families of primary fibrils were oriented in two directions at the model surface (+*x* and –*x* directions in Fig. 1a of the main text) (Mononen et al., 2012). The Cauchy stress tensor for the fibril *k* was defined as:

| $\boldsymbol{\sigma}_{f}^{k}=\left\{ \begin{matrix} \rho_{z}C\sigma_{f}\boldsymbol{e}_{f}\otimes\boldsymbol{e}_{f} for the primary fibrils \\ \rho_{z}\sigma_{f}\boldsymbol{e}_{f}\otimes\boldsymbol{e}_{f} for the secondary fibrils \end{matrix} \right.,$ | (S.11) |
| --- | --- |

where *C* is the ratio between primary and secondary fibrils density, $\rho_{z}$ is the depth-dependent relative collagen density and $\otimes$ denotes dyadic product.

Supplementary Table 1: Structural, compositional and material parameters for the fibril-reinforced poroviscoelastic swelling (FRPVES) material model of healthy bovine cartilage (Eskelinen et al., 2019; Tanska et al., 2019).

| Parameter | Value | Description |
| --- | --- | --- |
| Structural | | |
| $d_{sup}$ | 0.12 *h*^†^ | Superficial layer thickness |
| $d_{mid}$ | 0.26 *h* | Middle layer thickness |
| $d_{deep}$ | 0.62 *h* | Deep layer thickness |
| Compositional | | |
| $n_{f} (-)$ | 0.8 - 0.15*z*^‡^ | Depth-dependent fluid volume fraction |
| $\rho_{z} (-)$ | 1.4*z*^2^ - 1.1*z* + 0.59 | Depth-dependent relative collagen density |
| $c_{F,0} (mEq/ml)$ | 0.15 + 0.03*z* | Depth-dependent initial FCD content |
| Material | | |
| $C (-)$ | 12.16 | The ratio between primary and secondary fibrils density |
| $E_{0} (\mathrm{MPa})$ | 2.737 | Initial fibril network modulus |
| $E_{\varepsilon} (\mathrm{MPa})$ | 867.7 | Strain-dependent fibril network modulus |
| $\eta(MPa s)$ | 1418.0 | Viscoelastic damping coefficient |
| $E_{nf} (\mathrm{MPa})$ | 0.315 | Non-fibrilar matrix modulus |
| $\nu_{nf} (-)$ | 0.01 | Non-fibrilar matrix Poisson’s ratio |
| $k_{0} (m^{4}N^{-1}s^{-1})$ | 1.522 × 10^-15^ | Initial permeability |
| $M (-)$ | 1.339 | Permeability void-ratio dependency constant |
| ^†^ *h* denotes the total thickness of cartilage  ^‡^ *z* denotes the normalized thickness of cartilage from the surface (surface: *z* = 0, bottom: *z* = 1). | | |

The total stress tensor was estimated as:

| $\boldsymbol{\sigma}_{tot}=\boldsymbol{\sigma}_{nf}-\Delta\pi\boldsymbol{I}-T_{c}\boldsymbol{I}-\mu_{f}\boldsymbol{I}+\sum_{k=1}^{totf} \boldsymbol{\sigma}_{f}^{k}$ | (S.12) |
| --- | --- |

where $\mu_{f}$ is the chemical potential of water (Wilson et al., 2005b) and *totf* is the total number of fibril families (4 primary + 13 secondary = 17 fibril families). The structural, compositional and material parameters of the FRPVES model for healthy bovine articular cartilage were adopted from (Eskelinen et al., 2019; Tanska et al., 2019) and are given in Supplementary Table 1.

# References

Clark, J. M. (1985). The organization of collagen in cryofractured rabbit articular cartilage: A scanning electron microscopic study. *J. Orthop. Res.* 3, 17–29. doi:10.1002/jor.1100030102.

Eskelinen, A. S. A., Mononen, M. E., Venäläinen, M. S., Korhonen, R. K., and Tanska, P. (2019). Maximum shear strain-based algorithm can predict proteoglycan loss in damaged articular cartilage. *Biomech. Model. Mechanobiol.* 18, 753–778. doi:10.1007/s10237-018-01113-1.

Huyghe, J.M., Houben, G.B., Drost, M.R., and Van Donkelaar, C.C. (2003). An ionised/non-ionised dual porosity model of intervertebral disc tissue. *Biomech. Model. Mechanobiol.* 2, 3–19.

Meachim, G., Denham, D., Emery, I. H., and Wilkinson, P. H. (1974). Collagen alignments and artificial splits at the surface of human articular cartilage. *J. Anat.* 118, 101.

Mononen, M. E., Mikkola, M. T., Julkunen, P., Ojala, R., Nieminen, M. T., Jurvelin, J. S., et al. (2012). Effect of superficial collagen patterns and fibrillation of femoral articular cartilage on knee joint mechanics-a 3D finite element analysis. *J. Biomech.* 45, 579–587. doi:10.1016/j.jbiomech.2011.11.003.

Tanska, P., Julkunen, P., and Korhonen, R. K. (2018). A computational algorithm to simulate disorganization of collagen network in injured articular cartilage. *Biomech. Model. Mechanobiol.* 17, 689–699. doi:10.1007/s10237-017-0986-3.

Van der Voet, A. A. (1997). Comparison of finite element codes for the solution of biphasic poroelastic problems. *Proc. Inst. Mech. Eng. H.* 211, 209–211.

Wilson, W., van Donkelaar, C. C., van Rietbergen, B., and Huiskes, R. (2005). A fibril-reinforced poroviscoelastic swelling model for articular cartilage. *J. Biomech.* 38, 1195–1204. doi:10.1016/j.jbiomech.2004.07.003.

Wilson, W., van Donkelaar, C., and Huyghe, J. (2005). A comparison between mechano-electrochemical and biphasic swelling theories for soft hydrated tissues. *J. Biomech. Eng.* 127, 158–165.

Wilson, W., Huyghe, J. M., and Van Donkelaar, C. C. (2007). Depth-dependent compressive equilibrium properties of articular cartilage explained by its composition. *Biomech. Model. Mechanobiol.* 1, 43–53.
